# Magnesium Lithospermate B Attenuates High-Fat Diet-Induced Muscle Atrophy in C57BL/6J Mice

**DOI:** 10.3390/nu14010104

**Published:** 2021-12-27

**Authors:** Tsun-Li Cheng, Zi-Yun Lin, Keng-Ying Liao, Wei-Chi Huang, Cian-Fen Jhuo, Pin-Ho Pan, Chun-Jung Chen, Yu-Hsiang Kuan, Wen-Ying Chen

**Affiliations:** 1 Veterinary Medical Teaching Hospital, National Chung Hsing University, Taichung 402, Taiwan; flynstm@hotmail.com; 2 Department of Veterinary Medicine, National Chung Hsing University, Taichung 402, Taiwan; ad159632@gmail.com (Z.-Y.L.); emily93211@hotmail.com (K.-Y.L.); vh950420@yahoo.com.tw (W.-C.H.); pph.pgi@gmail.com (P.-H.P.); 3 Graduate Institute of Biotechnology, National Chung Hsing University, Taichung 402, Taiwan; quartz1248s@gmail.com; 4 Department of Pediatrics, Tungs’ Taichung Metro Harbor Hospital, Taichung 435, Taiwan; 5 Department of Medical Research, Taichung Veterans General Hospital, Taichung 407, Taiwan; cjchen@vghtc.gov.tw; 6 Department of Pharmacology, School of Medicine, Chung Shan Medical University, Taichung 402, Taiwan; kuanyh@csmu.edu.tw

**Keywords:** magnesium lithospermate B, muscle atrophy, obesity, insulin resistance, inflammation

## Abstract

Magnesium lithospermate B (MLB) is a primary hydrophilic component of Danshen, the dried root of *Salvia miltiorrhiza* used in traditional medicine, and its beneficial effects on obesity-associated metabolic abnormalities were reported in our previous study. The present study investigated the anti-muscle atrophy potential of MLB in mice with high-fat diet (HFD)-induced obesity. In addition to metabolic abnormalities, the HFD mice had a net loss of skeletal muscle weight and muscle fibers and high levels of muscle-specific ubiquitin E3 ligases, namely the muscle atrophy F-box (MAFbx) and muscle RING finger protein 1 (MuRF-1). MLB supplementation alleviated those health concerns. Parallel changes were revealed in high circulating tumor necrosis factor-α (TNF-α) and interleukin-6 (IL-6), skeletal TNF receptor I (TNFRI), nuclear factor-kappa light chain enhancer of activated B cells (NF-κB), p65 phosphorylation, and Forkhead box protein O1 (FoxO1) as well as low skeletal phosphoinositide 3-kinase (PI3K) and protein kinase B (Akt) phosphorylation. The study revealed that MLB prevented obesity-associated skeletal muscle atrophy, likely through the inhibition of MAFbx/MuRF-1-mediated muscular degradation. The activation of the PI3K-Akt-FoxO1 pathway and inhibition of the TNF-α/TNFRI/NF-κB pathway were assumed to be beneficial effects of MLB.

## 1. Introduction

Muscle atrophy occurs as a result of a net loss of muscle mass. In addition to its devastating effects on human health, muscle atrophy is a highly recognized risk factor for physical disabilities and a poor quality of life [1,2]. The majority of muscle mass depends on the counterbalance between protein anabolism and catabolism. Insufficient protein synthesis and overwhelmed protein degradation predispose people to muscle atrophy [3,4]. The prevalence of muscle atrophy is continually increasing because it is a complication of many acute and chronic diseases [5]. Therefore, a more comprehensive understanding of its pathogenic mechanism is necessary for the development of strategies to combat it and its associated sequelae.

Autophagy, caspases, and particularly the ubiquitin-proteasome system are three families of proteolytic enzymes common to protein degradation [6]. The core of the ubiquitin-proteasome system consists of three groups of enzymes, namely the ubiquitin E1 activating enzyme, ubiquitin E2 conjugating enzyme, and ubiquitin E3 ligase. Functional diversity and targeted specificity of the ubiquitin-proteasome system rely on the introduction of ubiquitin E3 ligase [7]. Muscle atrophy F-box (MAFbx)/atrogin-1 and muscle RING finger protein 1 (MuRF-1) are two muscle-specific ubiquitin E3 ligases that are highly expressed in the skeletal muscles under atrophy-prone conditions. Their transcriptional activation is closely linked with muscle atrophy independent of etiologies, and the inhibition of their expression reduces muscle mass loss [8]. Evidence has revealed the crucial roles of nuclear factor kappa light chain enhancer of activated B cells (NF-κB) and the Forkhead box protein O1 (FoxO) family transcription factor in the transcriptional activation of MAFbx and MuRF-1 and the consequences of muscle atrophy [5,8,9]. The phenomena underscore the roles of NF-κB and FoxO intervention as preventive or therapeutic options for patients with muscle atrophy.

Herbal plants are vital sources of biologically active compounds and are beneficial to patients with metabolic disorders [10]. Danshen, the root of the medicinal plant *Salvia miltiorrhiza*, is traditionally used to improve daily functioning and treat a wide variety of diseases, including coronary heart disease, hepatitis, menstrual disorders, blood circulation diseases, and cardiovascular diseases [11]. Magnesium lithospermate B (MLB), a derivative of a caffeic acid tetramer metabolized in the liver and excreted through the bile, is the primary hydrophilic component of Danshen [12]. It exerts pharmacological effects, such as anti-ischemia reperfusion, and contains antioxidant, anti-inflammatory, and antitumor properties [13,14,15]. Although the components of Danshen have indicated the promotion of muscle anabolism and prevention of muscle wasting [16,17], a study focused on MLB has not yet been conducted. MLB’s anti-inflammatory properties result from the inhibition of NF-κB [18], and MLB promotes Akt activity, a critical negative regulator of the FoxO family of transcription factors [19]. Its effects on NF-κB and Akt suggest that MLB may benefit the treatment of muscle atrophy.

Skeletal muscle atrophy is a common complication of obesity, a metabolic disease characterized by insulin resistance and inflammation, with poor outcomes [20,21,22]. Protein degradation appears to have a dominant role in obesity-associated skeletal muscle atrophy [23]. Previous studies revealed that MLB diminishes high-fat diet (HFD)-induced metabolic abnormalities through reducing obesity, fatty liver, glucose intolerance, and insulin resistance [24], and aging and obesity-induced ER stress, insulin resistance, and inflammasome formation in the liver [25]. To expand the study of MLB’s nutraceutical potential, we hypothesized that MLB may weaken skeletal muscle atrophy through acting on MAFbx and MuRF-1. To complete our working hypothesis, obesity-associated skeletal muscle atrophy was modeled in mice through an HFD and daily administrations of MLB.

## 2. Materials and Methods

### 2.1. Animals

Seven-week-old male C57BL/6J mice were purchased from BioLasco, Taiwan Co., Ltd. (Taipei, Taiwan). The mice were maintained on a 12:12 light–dark cycle with unrestricted access to regular food (5008 Rodent LabDiet, PMI Nutrition International Inc., St. Louis, MO, USA) and water. After a 1-week acclimation period, the mice were randomly divided into three groups. The control group (n = 8) consisted of mice that continued to eat regular food and the high-fat (HF) group (n = 8) comprised mice that ate high-fat food (high-fat Rodent TestDiet, PMI Nutrition International Inc., St. Louis, MO, USA); 67% of calories were obtained from fat. The HF + MLB group (n = 8) consisted of mice that consumed HF food and daily MLB supplements (100 mg/kg body weight/day) that were dissolved in water and administrated through oral gavage. MLB, purified from *S. miltiorrhiza*, was purchased from KO DA Pharmaceutical Co., Ltd. (Taoyuan, Taiwan) with a purity of approximately 85%.

### 2.2. Fasting Blood Glucose Measurement

After 17 weeks of supplementation, the mice fasted overnight. A blood droplet from a tail clipping and a glucometer with test strips (Roche Ltd., Basel, Switzerland) were used to measure each mouse’s fasting blood glucose level.

### 2.3. Serum Analyses

Blood samples were collected through cardiac puncture and were centrifuged at 3000 rpm for 10 min to separate the serum. The serum levels of aspartate transaminase (AST), alanine transaminase (ALT), total cholesterol, and triacylglycerol were measured using a clinical chemistry analyzer (Hitachi Autoanalyzer 7070, Hitachi Ltd., Tokyo, Japan). The levels of insulin (Crystal Chem Inc., Elk Grove Village, Illinois, USA), tumor necrosis factor-α (TNF-α), and interleukin-6 (IL-6; Quantikine R&D Systems, Minneapolis, MN, USA) were measured using enzyme-linked immunosorbent assay (ELISA) kits, following the procedures provided by the respective manufacturers.

### 2.4. HOMA-IR

HOMA-IR, a method developed by Matthews et al. (1985) [26], is an index used to quantify insulin resistance. The index is calculated as follows: HOMA-IR = fasting glucose (mg/dL) × serum insulin (mU/L)/405.

### 2.5. Histological Examination

At the end of the 17-week experimental period, the mice were euthanized under anesthesia with Zoletil 50 (40 mg/kg, IP; Virbac Laboratories, Carros, France), and the epididymal fat and gastrocnemius (GC), extensor digitorum longus (EDL), and soleus (SOL) muscles were quickly removed and weighed. The resected tissues, namely the epididymal fat and GC muscles, were fixed in 10% formalin and embedded with paraffin. Hematoxylin and eosin (H&E) staining was performed according to standard procedures. Histological images were captured with a light microscope (Olympus, BX43, Tokyo, Japan) equipped with a digital camera (Canon EOS 600 D, Tokyo, Japan). The H&E-stained sections were used for cross-sectional area (CSA) analyses. The numbers of adipocytes in the epididymal adipose tissues and the numbers of fibers in the GC muscles were counted in a total number of 100 per animal. Semiautomatic quantification of the minimal Feret’s diameter of fibers was performed as described previously [27]. The variance coefficient of minimal Feret’s diameter from each section was defined to evaluate the muscle fiber size variability among the groups and then calculated as the ratio between the standard deviation (×1000) and the mean of the diameter and was expressed as arbitrary units.

### 2.6. Tissue Preparation and Western Blot Analysis

The GC muscle tissues were homogenized with a lysis buffer (1% Triton X-100; 50 mM Tris-HCl, pH 7.6; 150 mM NaCl) and 1% protease inhibitor cocktail for protein extraction. The obtained proteins were separated through sodium dodecyl sulphate-polyacrylamide gel electrophoresis (SDS-PAGE) and electrophoretically transferred to polyvinylidene difluoride membranes. The blots were then incubated with antibodies, namely phosphatidylinositol 3-kinase (PI3K) p85g (Cell Signaling Technology, Inc., Danvers, MA, USA), protein kinase B (Akt; Thermo Fisher Scientific, Rockford, IL, USA), phospho-Akt (ser473; Cell Signaling Technology, Inc., Danvers, MA, USA), MAFbx/Atrogin-1 (Santa Cruz Biotechnology, Dallas, TX, USA), MURF-1 (Santa Cruz Biotechnology, Dallas, USA), FOXO1 (Proteintech, Rosemont, USA), phospho-FOXO1 (Cell Signaling Technology, Inc., Danvers, MA, USA), TNF-α (PeproTech, East Windsor, Mercer County, NJ, USA), TNFRI (Santa Cruz Biotechnology, Dallas, TX, USA), NF-κB p65 (Santa Cruz Biotechnology, Dallas, TX, USA), phospho-NF-κB p65 (Santa Cruz Biotechnology, Dallas, TX, USA), IL-6 (Santa Cruz Biotechnology, Dallas, TX, USA), and GAPDH (Santa Cruz Biotechnology, Dallas, TX, USA). After incubation with horseradish peroxidase-labeled IgG, the blots were developed using ECL Western blotting reagents and quantified with optical densitometry (Image Master ID) of the developed autoradiographs.

### 2.7. RNA Isolation and Quantitative Real-Time Reverse Transcriptase Polymerase Chain Reaction (RT-qPCR)

Total RNAs were isolated from excised epididymal adipose tissues using a TriZol RNA isolation reagent (Invitrogen, Carlsbad, CA, USA) and subjected to conventional cDNA synthesis and real-time PCR. The C_t_ values obtained were used to calculate relative levels of gene expression based on the ΔΔCT method. Oligonucleotides for PCR were: TNF-α, 5′-TCCCAACAAGGAGGAGAAGT and 5′-TGGTATGAAGTGGCAAATCG; IL-6, 5′-AGGTTCCATGTGCAAGTGTCT and 5′-GACAGCCCTGGTCAAAGGTT, and β-actin, 5′-AGAGGGAAATCGTGCGTGAC, and 5′-CAATAGTGATGACCTGGCCGT.

### 2.8. Statistical Analyses

One-way analysis of variance (ANOVA) was used for statistical analysis, and the differences between the three groups were determined using Bonferroni’s post hoc test (SPSS Statistics version 22). The results were deemed statistically significant at *p* < 0.05. All data are presented as the mean ± SD.

## 3. Results

### 3.1. MLB Supplementation Attenuated HFD-Induced Adiposity

**Figure 1:**
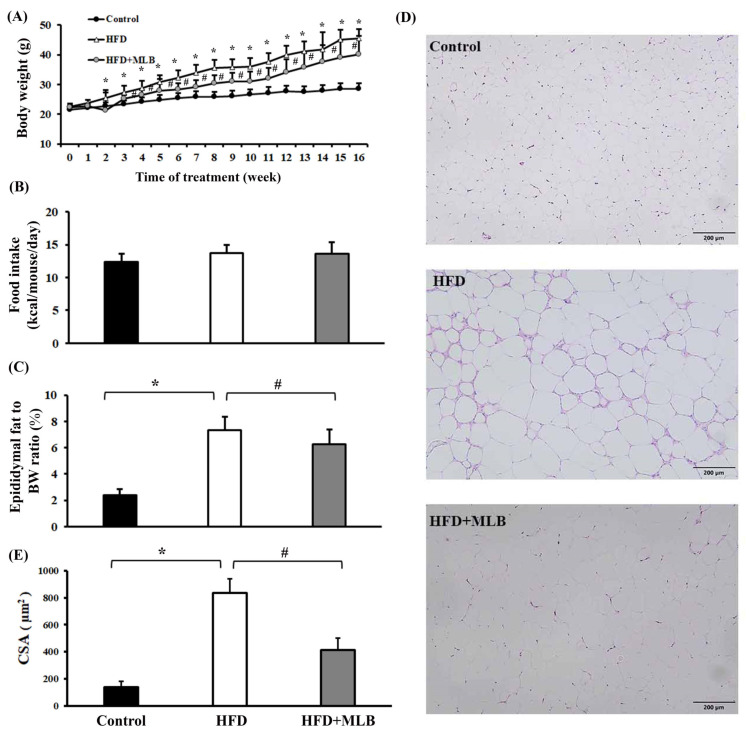
MLB supplementation attenuated HFD-induced adiposity. Over the course of 16 weeks, the mice’s body weight and food intake were recorded. Weekly changes in body weight (**A**) and food intake (**B**) are depicted and summarized. At the end of the experiment, the excised epididymal adipose tissues were weighed (**C**) and subjected to histological examination. Representative photomicrographs of H&E stain are displayed (**D**) and the relative epididymal adipose cross-sectional area (CSA) data are illustrated (**E**). * *p* < 0.05 vs. control group. # *p* < 0.05 vs. HFD group. n = 8. Scale bar: 200 μm.

The results revealed that an HFD increased the mice’s weight (Figure 1A), but had no clear effect on their average food intake (Figure 1B), compared with the mice that were fed a regular diet. Daily MLB supplementation limited the mice’s weight gain (Figure 1A). The percentage of the epididymal fat weight of the total weight of the HFD mice was significantly higher than that of the control group (Figure 1C). MLB supplementation reduced the HFD-induced increase in epididymal fat weight (Figure 1C). H&E staining revealed larger adipocytes (Figure 1D), macrophage infiltration, and increased CSAs (Figure 1D,E) in the epididymal fat pads of the HFD mice compared with those in the control group, and MLB supplementation decreased the size of the adipocytes in the HFD mice. These results suggested that MLB supplementation alleviated HFD-induced adiposity.

### 3.2. MLB Supplementation Reduced HFD-Induced Insulin Resistance, Dyslipidemia, and Liver Injury

**Figure 2:**
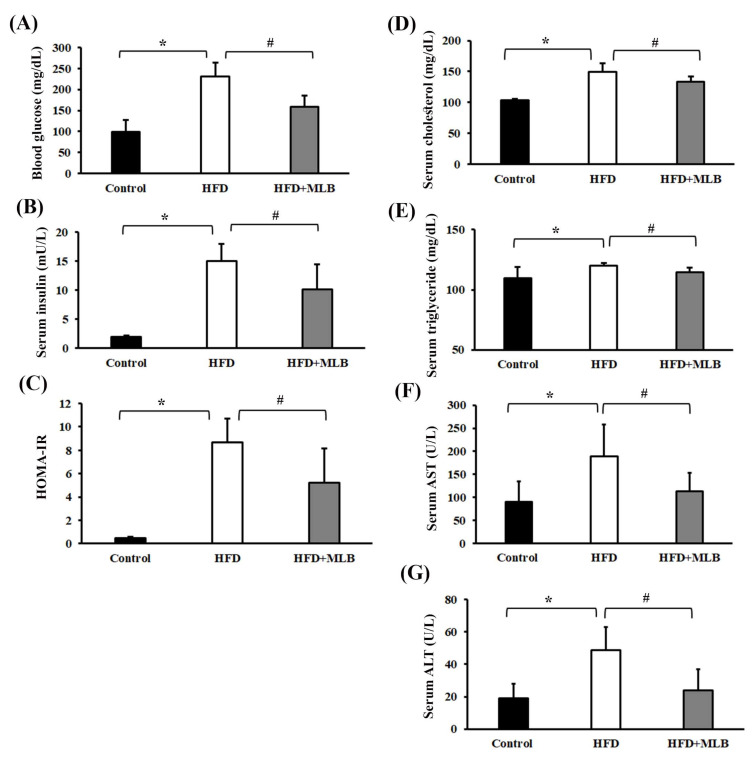
MLB supplementation improved HFD-induced insulin resistance, dyslipidemia, and liver injury. At the end of the experiment, serum samples were subjected to analyses to measure fasting glucose (**A**), insulin (**B**), cholesterol (**D**), triglycerides (**E**), AST (**F**), and ALT (**G**). The value of HOMA-IR is depicted in (**C**). * *p* < 0.05 vs. control group. # *p* < 0.05 vs. HFD group. n = 8.

Several parameters reflecting the insulin action, lipid profile, and liver function were assessed to examine the effects of MLB on insulin resistance, lipid metabolism, and liver injury. At the end of the 17-week experiment, the HFD mice exhibited increased levels of fasting blood glucose, serum insulin, serum cholesterol, serum triglycerides, serum AST and serum ALT, all of which were alleviated by MLB supplementation (Figure 2A,B,D–G), indicating that MLB can induce hypoglycemia, hypoinsulinemia, and hypolipidemia, and improve liver function. A parallel HOMA-IR assessment indicated that the HFD mice exhibited impaired insulin sensitivity, and MLB supplementation reduced their HFD-induced insulin resistance (Figure 2C). These results indicated that MLB supplementation may have reduced hyperglycemia, hyperinsulinemia, insulin resistance, dyslipidemia, and liver injury in HFD mice.

### 3.3. MLB Supplementation Reduced HFD-Increased Proinflammatory Cytokines

**Figure 3:**
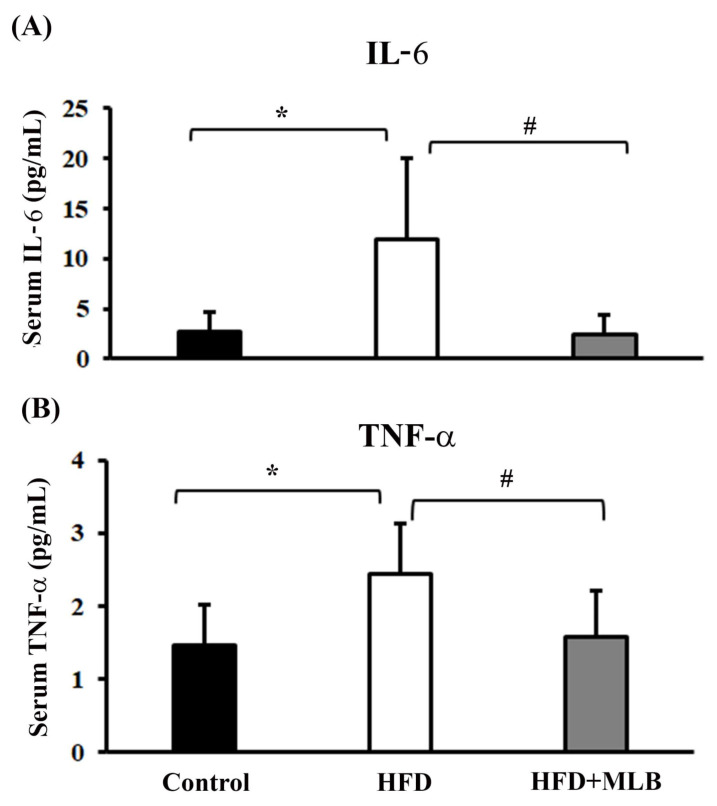
MLB supplementation reduced HFD-induced proinflammatory cytokines. At the end of the experiment, serum samples were subjected to measurements of IL-6 (**A**) and TNF-α (**B**) with commercially available enzyme immunosorbent assay kits. * *p* < 0.05 vs. control group. # *p* < 0.05 vs. HFD group. n = 8.

Inflammation is a result of muscle wasting, and an elevated expression of proinflammatory cytokines correlates with several clinical disorders, including obesity and insulin resistance [28,29]. In our study, the ELISA data illustrated the effects of MLB supplementation on IL-6 and TNF-α levels in the serum of mice with HFD-induced obesity. A significant increase was noted in the IL-6 and TNF-α levels in the serum of the HFD mice compared with the control group (Figure 3A,B). MLB supplementation significantly decreased the production of HFD-induced IL-6 and TNF-α (Figure 3A,B). These findings revealed that MLB supplementation could attenuate an inflammatory response in mice with HFD-induced obesity by lowering the levels of circulating proinflammatory cytokines.

### 3.4. MLB Supplementation Attenuated HFD-Induced Muscle Atrophy

**Figure 4:**
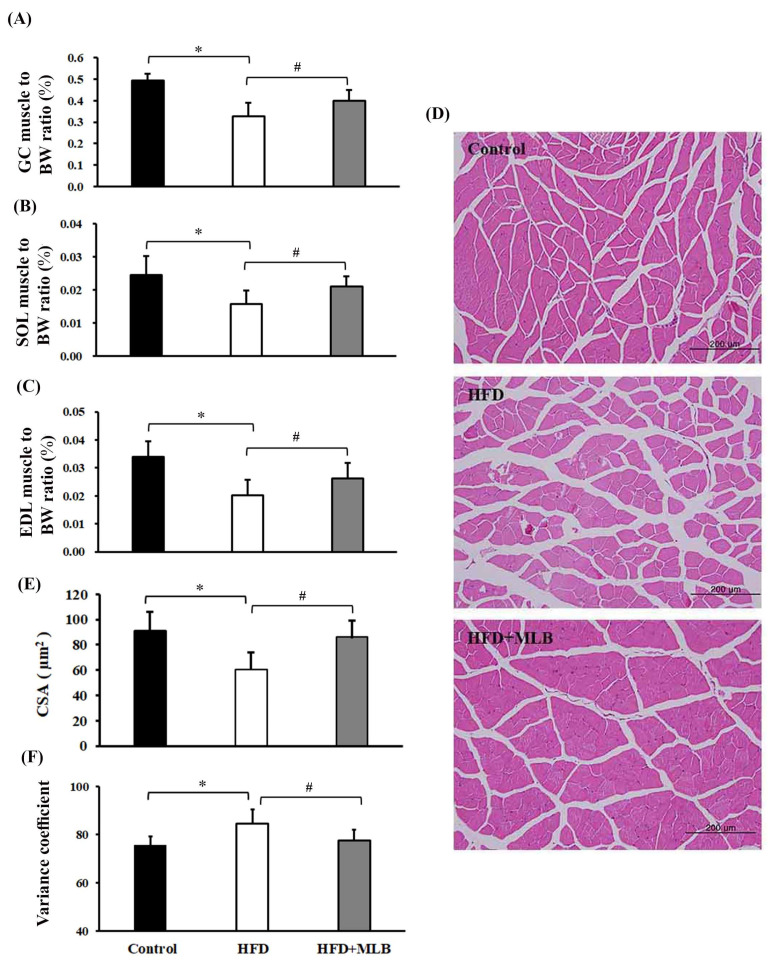
MLB supplementation attenuated HFD-induced muscle atrophy. At the end of the experiment, the skeletal muscles, namely the gastrocnemius (GC), extensor digitorum longus (EDL), and soleus (SOL) muscles, were removed and weighed. The ratios of the weight of the GC, SOL, and EDL muscles to the body weight are depicted (**A**–**C**). The representative hematoxylin and eosin (H&E) staining of GC muscles from each group are presented (**D**), and the relative muscle cross-sectional area (CSA) data are illustrated (**E**). Measurement of variance coefficients of the fiber size in the cross-sectional samples of three groups using Feret’s diameter as the geometrical parameter (**F**). Values are expressed as the mean ± SD. * *p* < 0.05 vs. control group. # *p* < 0.05 vs. HFD group. n = 8.

Skeletal muscle atrophy is a common complication of obesity [30,31]. The potential effects of MLB on skeletal muscle atrophy were investigated in mice that had HFD-induced obesity. The percentage of the weights of the GC (Figure 4A), SOL (Figure 4B), and EDL muscles (Figure 4C) of total body weight decreased in the HFD mice. Histological examination revealed a reduced CSA and an increased variance coefficient of the GC muscle fibers (Figure 4D–F) in the HFD mice compared with the other groups of mice. Those muscular changes were alleviated by MLB supplementation (Figure 4), which, according to the results, could attenuate HFD-induced muscle atrophy.

### 3.5. MLB Supplementation Attenuated HFD-Increased E3 Ligases

**Figure 5:**
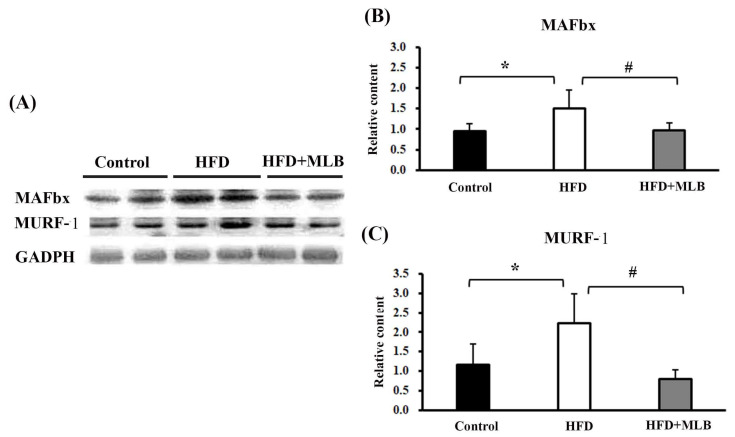
MLB supplementation attenuated HFD-induced muscle atrophy through inhibiting protein degradation in skeletal muscles. Proteins were isolated from skeletal muscles and subjected to a Western blot with antibodies against MAFbx, MURF-1, and GAPDH. Two representative samples from each group are presented (**A**), and the quantitative data are depicted (**B**,**C**). Values are expressed as the mean ± SD. * *p* < 0.05 vs. control group. # *p* < 0.05 vs. HFD group. n = 8.

To further examine the effects of MLB on HFD-induced muscle atrophy, we investigated the changes in the muscle-specific ubiquitin E3 ligases in the mice’s skeletal muscles. The representative blots of the Western blot analysis are presented in Figure 5A. Quantitative results revealed that an HFD significantly increased the expression of MAFbx (Figure 5B) and MuRF-1 (Figure 5C), and the changes were attenuated by MLB supplementation. The results indicated that MLB attenuated muscle atrophy in HFD mice, likely through the inhibition of the ubiquitin E3 ligase expression.

### 3.6. MLB Supplementation Attenuated HFD-Activated FoxO1 Signaling

**Figure 6:**
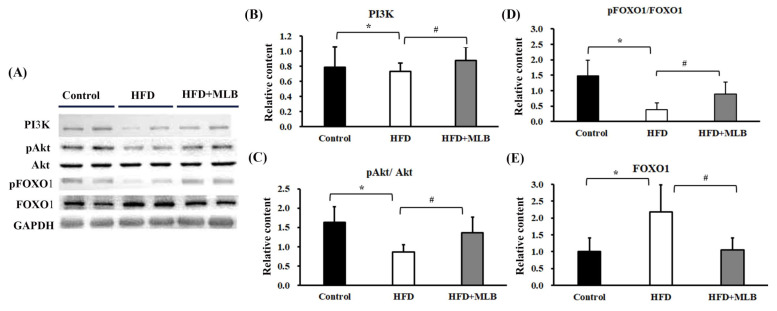
MLB supplementation attenuated HFD-induced muscle atrophy through the upregulation of PI3K/Akt/FOXO1 signaling. Proteins were isolated from skeletal muscles and subjected to a Western blot with antibodies against PI3K, pAkt, Akt, pFOXO1, FOXO1, and GAPDH. Two representative samples from each group are presented (**A**), and the quantitative data are depicted (**B**–**E**). Values are expressed as the mean ± SD. * *p* < 0.05 vs. control group. # *p* < 0.05 vs. HFD group. n = 8.

The PI3K/Akt/FoxO axis signaling played a substantial role in the regulation of the MAFbx and MuRF-1 expression [3]. The HFD mice exhibited decreased PI3K protein content (Figure 6A,B), Akt phosphorylation (Figure 6A,C), and FoxO1 phosphorylation (Figure 6A,D), and increased FoxO1 protein content (Figure 6A,E) in their skeletal muscles. The changes in the PI3K/Akt/FoxO signaling molecules were attenuated through MLB supplementation (Figure 6). The results indicated that MLB attenuated HFD-induced FoxO1 activation through the promotion of Akt signaling.

### 3.7. MLB Supplementation Attenuated HDF-Activated TNF-α/NF-κB Signaling

**Figure 7:**
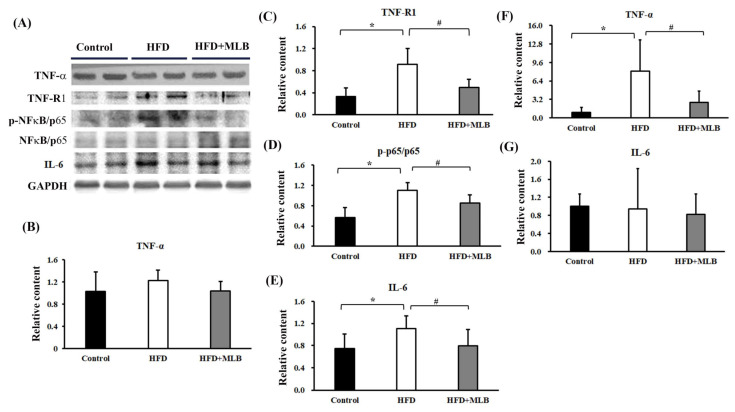
MLB supplementation attenuated HFD-induced muscle atrophy through the downregulation of TNF-α/NF-κB signaling. Proteins were isolated from the skeletal muscles and subjected to a Western blot with antibodies against TNF-α, TNFRI, NF-κB/p65, p-NF-κB/p65, IL-6, and GAPDH. Two representative samples from each group are presented (**A**), and the quantitative data are depicted (**B**–**E**). Total RNAs were extracted from epididymal adipose tissues and subjected to RT-qPCR for the measurement of TNF-α (**F**) and IL-6 (**G**) mRNA levels. Values are expressed as mean ± SD. * *p* < 0.05 vs. control group. # *p* < 0.05 vs. HFD group. n = 8.

TNF-α is a cachectic factor that induces MAFbx and MuRF-1 expressions, which leads to the induction of muscle atrophy in an NF-κB-dependent mechanism [32,33]. Although the TNF-α levels were not significantly altered (Figure 7A,B) in the skeletal muscles of the HFD mice, an elevated TNF-RI protein content (Figure 7A,C) and p65 phosphorylation (Figure 7A,D) were noted. An elevated expression of IL-6 protein was also revealed (Figure 7A,E). Intriguingly, HFD mice elevated TNF-α mRNA expression (Figure 7F) in the epididymal adipose tissues, while having little effect on IL-6 mRNA (Figure 7G). Those changes were attenuated by MLB supplementation (Figure 7). The results indicated that MLB attenuated HFD-activated TNF-α/NF-κB and IL-6 signaling through different modes of action.

## 4. Discussion

MLB reduced obesity, aging, and diabetes by reducing hepatic inflammation and insulin resistance [24,25]. This study demonstrated that MLB supplementation attenuated obesity-associated skeletal muscle atrophy in HFD mice through the regulation of the PI3K/Akt/FoxO1 and TNF-α/NF-κB signaling pathways, leading to the inhibition of muscle-specific ubiquitin E3 ligase expression.

Muscle atrophy is a serious health concern, and the majority of the skeletal muscular protein structure is governed by protein synthesis and degradation. Under insufficient nutrition or malnutrition, the impaired IGF-1-mediated macromolecular synthesis creates an obstacle to muscle mass maintenance. However, abnormal protein degradation has caused most cases of muscle atrophy [34]. Consistent with relevant studies [35,36], the HFD mice exhibited signs of skeletal muscle atrophy with concurrent activation of MAFbx and MuRF-1. This study presented the first evidence that daily MLB supplementation diminished skeletal muscle atrophy, and the improvement correlated with the reduction in skeletal MAFbx and MuRF-1 expression. The results revealed that the ubiquitin-proteasome system played a substantial role in obesity-associated skeletal muscle atrophy, and MLB had an anti-atrophy effect through negatively affecting skeletal MAFbx and MuRF-1 expression.

The etiologies of muscle atrophy are multifactorial. Obese people often develop muscle atrophy [22,23], and those with excessive caloric intakes and deposition of fat are prone to develop insulin resistance and chronic inflammation [20,21]. At the molecular level, obesity is associated with impaired Akt activity, enhanced NF-κB and FoxO1, and increased TNF-α and IL-6 levels [37], all of which are related to muscle atrophy [37]. We reported that MLB reduced obesity, fatty liver, glucose intolerance, and insulin resistance in HFD mice [24]. In this study, the HFD mice that received MLB supplements exhibited activated skeletal Akt, inhibited skeletal NF-κB and FoxO1, and reduced circulating TNF-α and IL-6. Thus, MLB contains diverse pharmacological properties that are attributed to diminished obesity-associated biochemical and molecular changes.

The FoxO family transcription factors have integrative roles in glucose and lipid metabolism. FoxO1, a typical FoxO family member, regulates hepatic gluconeogenesis, glycogenolysis, and lipogenesis in response to the insulin signal. The insulin-PI3K-Akt pathway negatively regulates FoxO1 transcription factors. Conversely, decreased PI3K-Akt activity prevents the degradation of FoxO1 and enforces FoxO1 transcriptional activity [3]. Furthermore, FoxO1 actively participates in the transcriptional activation of MAFbx and MuRF-1 expression [3]. An inhibited PI3K-Akt pathway and FoxO1 phosphorylation and increased FoxO1 protein content were observed in the skeletal muscles of the HFD mice, and MLB reversed the inactivation of the PI3K-Akt pathway. MLB’s improvement of the HFD mice’s insulin resistance strongly suggested that the restoration of the PI3K-Akt pathway not only played a role in glucose metabolism but also had an inhibitory effect on the FoxO1/MAFbx and MuRF-1 pathways and reduced obesity-associated muscle atrophy.

Skeletal muscle atrophy is highly associated with inflammatory responses and cytokines [38,39]; particularly, TNF-α is a recognized cachetic factor [40,41]. Evidence indicated that the tissue expressions of MAFbx and MuRF-1 messenger ribonucleic acid (mRNA) correlated with elevated TNF-α levels in an inflammatory catabolic state [42,43]. The presence of TNF-α promoted MAFbx/MuRF-1 mRNA expression, in vivo and in vitro [44,45]. TNF-α increased the effectiveness of the ubiquitin-proteasome pathway and caused muscular degradation in the C2C12 myotubes and skeletal muscles through the activation of NF-κB signaling [32,33]. By contrast, anti-TNF treatments reduced rat skeletal muscle wasting [40]. Upon activation, TNF-α transduced intracellular signals through engagement with TNF Receptor I (TNFRI) and converged onto NF-κB [40,41]. In this study, the HFD mice exhibited an increased circulating TNF-α level, adipose TNF-α mRNA level, skeletal TNFRI expression, and skeletal NF-κB p65 phosphorylation. Parallel elevation was demonstrated in the skeletal MAFbx and MuRF-1 expressions and muscle atrophy, and MLB supplementation alleviated those TNF-α-related inflammatory changes. Therefore, MLB’s inhibition of the TNF-α/TNFRI/NF-κB pathway represented an alternative means of decreasing the MAFbx and MuRF-1 expression and muscle atrophy. However, the levels of skeletal TNF-α remained consistent with no remarkable change among groups. Moreover, MLB alleviated metabolic abnormality and hepatic inflammation in obese mice [24,25]. All aforementioned data indicate that the anti-atrophy effect of MLB is probably secondary to the reduction in tissue inflammation, such as hepatic and adipose tissues.

## 5. Conclusions

This study revealed that daily MLB supplements reduced obesity-associated skeletal muscle atrophy, likely through the inhibition of MuRF-1- and MAFbx-mediated muscular degradation. The activation of the PI3K-Akt-FoxO1 pathway and inhibition of the TNF-α/TNFRI/NF-κB pathway were assumed to be attributed to MLB. In addition to improving obesity-associated metabolic abnormalities, as reported in our previous study [24], the data presented in this study further highlights MLB’s potential to combat obesity-associated muscle atrophy.

## Data Availability

The authors confirm that the data supporting the findings of this study are available within the article.
